# Health-related quality of life and associated factors among people living with HIV/AIDS in Lagos, Nigeria

**DOI:** 10.4102/phcfm.v16i1.4519

**Published:** 2024-08-30

**Authors:** Temitope S. Oladejo, Hellen Myezwa, Adedayo T. Ajidahun, Sam Ibeneme

**Affiliations:** 1 Department of Physiotherapy, Faculty of Health Sciences, University of the Witwatersrand, Johannesburg, South Africa; 2 School of Therapeutic Sciences, Faculty of Health Sciences, University of the Witwatersrand, Johannesburg, South Africa; 3 Department of Medical Rehabilitation, Faculty of Health Sciences, College of Medicine, University of Nigeria, Enugu, Nigeria

**Keywords:** health-related quality of life, HIV, Nigeria, associated factors, anti-retroviral therapy

## Abstract

**Background:**

Although people living with HIV (PLWH) now have a longer life expectancy due to antiretroviral therapy, several factors impact their health-related quality of life (HRQoL). Understanding the dimensions and determinants of HRQoL among PLWH is crucial to developing solutions to improve their overall wellbeing.

**Aim:**

This research aimed to explore the HRQoL and its associated factors among PLWH in Lagos, Nigeria.

**Setting:**

Seven HIV testing and treatment centres in Lagos.

**Methods:**

A cross-sectional survey was conducted with 385 participants. Socio-demographic and HRQoL data were obtained using questionnaires and the Medical Outcomes Study HIV Health Survey (MOS-HIV). Logistic regression models were used to identify variables that were associated with quality of life.

**Results:**

The physical health summary and mental health summary scores measured by the MOS-HIV were 54.2 ± 5.3 and 56.3 ± 6.7, respectively. Being married, having higher levels of education, shorter duration of HIV and higher income levels were significantly associated with better HRQoL. The duration of HIV was found to have an inversely proportional influence on the quality of life of PLWH, both in physical health (χ^2^ = 9.477, *p* = 0.009) and mental health (χ^2^ = 11.88, *p* = 0.004) dimensions.

**Conclusion:**

The HRQoL of PLWH in Lagos, Nigeria was relatively low. Education, duration of HIV, marital status and income level are predictors of HRQoL.

**Contribution:**

This study is valuable for healthcare professionals and policymakers, providing them with essential information to tailor interventions and allocate resources effectively to improve the overall wellbeing of PLWH in Nigeria.

## Introduction

Of the estimated 37.9 million persons with HIV (PLWH) living in the world, 67.5% are in Africa.^1^ Despite the low prevalence of 2.1% (1.9 million PLWH), Nigeria has the second-largest population of PLWH in the world.^2^ Although there has been an increase in access to Anti-Retroviral Therapy (ART) in Nigeria, the rate of new infections and opportunistic infections, such as tuberculosis, has risen in the past year.^1^ This indicates that although mortality rates among PLWH may fall, they are, however, prone to a diverse range of health-related challenges because of the HIV infection and their use of ART.

The Federal Government of Nigeria led by National Agency for the Control of AIDS (NACA) in the recent National HIV/AIDS strategic framework 2021–2025,^3^ emphasised the aim for Nigeria to be AIDS-free by 2030, with no new infections, discrimination, or deaths because of the disease. One of the seven principles guiding this strategic framework is ‘to ensure care and support for all people living with and affected by HIV’.^4^ Central to this target is the UNAIDS 95-95-95 goal of ensuring that, 95% of persons living with HIV are aware of their HIV status, 95% of patients diagnosed with HIV undergo ongoing antiretroviral medication and 95% of people on antiretroviral medication maintain viral suppression.^5^ Currently, these strategies have aided in transforming HIV/AIDS from a deathly acute infection to a chronic disease, as PLWH are living longer lives because of improved access to ART.^6^ Thus, these strategies collectively aim at improving the health-related quality of life (HRQoL) of PLWH.

The definition of HRQoL continues to be debatable. According to the World Health Organization, quality of life is defined as ‘the individual’s perception of their position in the context of culture and value systems in which they live and in relation to their goals, expectations, standards and concerns’.^7^ This concept renders quality of life (QoL) subjective and specific to a person, his or her culture, and environment. Although an individual’s personal view about their quality of life may alternate throughout their life,^8^ the multidimensional concept of HRQoL encompasses various domains such as emotional health, physical health, social functioning (SF), and pain, which play a vital role in how an individual rates their overall perception of general health.^9,10^ In furtherance of this, various factors that could impact the HRQoL of a PLWH have been explored,^11^ and findings reveal that sociodemographic characteristics, presence of comorbidities, socioeconomic status, environmental factors, coping mechanisms, and clinical factors could influence the HRQoL of PLWH.

Studies exploring the influence of various factors in determining the QoL of PLWH have been assessed nationally^12,13^ and globally.^14,15,16^ Findings from these studies reveal that the determinant factors vary and range across physical, psychological, social and financial domains.^17,18^ These factors also differ across participants based on geographical locations, ethnicity, gender, and other concurrent factors.^11^

Several instruments have been utilised in measuring the HRQoL of PLWH.^19^ Considering the diverse instruments employed to assess the HRQoL of PLWH, including widely used measures such as the Medical Outcomes Study HIV Health Survey (MOS-HIV) and the World Health Organization Quality of Life (WHOQOL)-BREF,^20^ the literature on HRQoL among PLWH in Nigeria lacks consensus. While Ogbuji and Oke^21^ utilised the ‘HIV Symptom Scale’ (HSS) and the ‘Quality of Life Scale’ (QOLS) to report poor quality of life in Ibadan, Nigeria, a study in South-East Nigeria using the WHOQOL brief version tool indicated an overall good HRQoL among adolescents and adults.^22^ Similarly, Salako et al.^23^ 2022 assessed HRQoL in Lagos, focusing on children and adolescents with the Paediatric Quality of Life Inventory [PedQoL™] and reported favourable HRQoL scores. This divergence in findings underscores the need for a comprehensive understanding of the factors influencing HRQoL in the Nigerian context.

A scoping analysis of HIV/AIDS in Nigeria^24^ from 1986 to 2021 surmised that although there is an increase in scientific literature pertaining to HIV/AIDS-related research in Nigeria, there are still unexplored grey areas, such as the relationship between sociodemographic status and quality of life among PLWH in ‘key population hotspots’^25^ which had not been precisely characterised. Understanding the factors impacting the HRQoL among PLWH is crucial for identifying potential confounding variables and understanding how different demographic factors may influence the HRQoL of PLWH. Additionally, sociodemographic data can provide insights into the distribution of health outcomes among PLWH which may assist policymakers to allocate resources effectively and implement policies that address the distinct needs and challenges encountered by PLWH.^26^ Hence, the primary objective of this research is to assess the current status of HRQoL and identify the factors associated with it among PLWH in Lagos, Nigeria, using the MOS-HIV questionnaire.

## Research methods and design

### Study design and participants

This cross-sectional study was conducted between July 2022 and January 2023 in Lagos, Nigeria. With Lagos being one of the key population hotspots for targeted HIV programme planning in Nigeria,^25^ seven HIV/AIDS testing and treatment centres in Lagos were randomly selected as study sites for the survey. The required sample size was determined using relevant literature^22,27^ and the Cochran’s sample size equation for categorical data^28^:


n=(t)2(p)(q)÷(d)2 [Eqn 1]

where:

*t* = value for selected alpha level of 0.025 in each tail = 1.96;

*d* = acceptable margin of error = 5%; and

(*p*)(*q*) is the estimate of variance = 0.25.

Thus, a minimum sample size of 385 participants was calculated. Participants were selected using convenience sampling, and the following inclusion criteria were applied in determining the eligibility of participants:

- people living with HIV;
- people above the age of 18;
- people who voluntarily indicated interest to participate and are able to consent.

### Data collection

Data collection was done using paper questionnaires. The principal investigator was assisted by two physiotherapists who worked as research assistants for the study. All researchers underwent training on administering the questionnaires and provided information to participants about the study’s purpose, content and potential risks before data collection. Participants completed the anonymous questionnaires independently while the researchers provided detailed clarification and recorded answers for participants who had difficulty understanding or reading the questionnaire.

### Measurements

#### Sociodemographic characteristics

The sociodemographic survey tool used in this study provided information on the age, gender, educational qualifications, income range, duration since HIV diagnosis, and ART information of participants.

#### Medical symptoms

Based on literature and clinical experience, common symptoms experienced by PLWH were included in the questionnaire. This questionnaire asked participants to respond ‘yes’ or ‘no’ to symptoms they had experienced over the previous 14 days.

#### Health-related quality of life

The MOS-HIV was utilised as the primary measure of HRQoL. Medical Outcomes Study HIV Health Survey is a widely used 35-item questionnaire that comprehensively assesses various dimensions of health relevant to HIV/AIDS,^29^ including general health perceptions (GHP), pain, physical functioning (PF), role functioning, SF, mental health (MH), energy, fatigue, cognitive function, and overall quality of life. Previous studies have reported satisfactory reliability, with a Cronbach’s α coefficient exceeding 0.7 for group comparisons,^30^ indicating adequate internal consistency. Additionally, validity has been established for the physical health summary (PHS) and mental health summary (MHS) scores of the MOS-HIV.^30^ Scoring of the questionnaire involved a two-step process: firstly, numerical values were re-coded, and then each item was scored on a scale of 0 to 100, with higher scores reflecting better functioning and overall well-being. Secondly, to estimate the 10 domains of patient functioning, items from the same scale were averaged.

### Statistical analyses

The data from the completed questionnaires were captured in Microsoft Excel and imported into IBM SPSS 25 ^®^ for analysis. Descriptive statistics were utilised to summarise all demographic variables. For the quantitative measures, continuous variables were presented as mean ± standard deviation (M ± s.d.) while categorical variables were described using percentages. Using the MOS-HIV scoring protocol,^30^ the individual scores from the questionnaire were re-coded where required and computed to achieve domain scores (PF, pain, SF, role functioning, emotional well-being, energy, fatigue, cognitive function, health distress (HD), health transition, general health, and overall quality of life) with a mean of 50, and a standard deviation of 10. Scores above 50 indicated better HRQoL, while scores below 50 indicated poorer HRQoL.

Univariate analysis using the χ^2^-test for categorical data and Spearman’s correlation for continuous data were utilised to assess the quality of life among PLWH across the various sociodemographic characteristics. Fischer’s Exact Test was utilised to ascertain the relationship between HRQoL and sociodemographic variables. Binary logistic regression analysis was conducted to identify factors associated with HRQoL in PLWH. The goodness-of-fit of the regression model was evaluated using the Hosmer–Lemeshow statistic, with a *p*-value > 0.05 indicating a well-fitting model. All variables with a significance level of *p* ≤ 0.2 in the univariate analysis were included in a multivariable logistic regression analysis.

### Ethical considerations

Approval was obtained from the Human Research Ethics Committee of the University of the Witwatersrand (M200906) and the Lagos State University Teaching Hospital Health Research Ethics Committee (LREC/06/10/1547). The clinical director of the participating testing and treatment centres granted permission. Written informed consent was obtained from the participants.

## Results

### Sociodemographic characteristics of participants

A total of 385 PLWH participated in the study, of which 27% (104) were male and 73% (281) were female. Most participants (66.5%) were between the ages of 31 and 50 years, and the overall mean (s.d.) age was 42.22 ± 10.43 years. Over half (*n* = 219; 56.9%) of the participants were married and had a secondary education (*n* = 194; 50.4%). Majority of the participants were low and middle-income earners, as only 12.7% (*n* = 49) of the participants earned above ₦70 000 ($76.81) per month. The sociodemographic characteristics of the participants are presented in Table 1.

**TABLE 1:** Sociodemographic characteristics of the people living with HIV in Lagos, Nigeria (*N* = 385).

Variables	*N*	%
**Age**		
18–30 years	48	12.5
31–50 years	256	66.5
> 51	81	21.0
**Gender**		
Male	104	27.0
Female	281	73.0
**Marital status**		
Married	219	56.9
Single	101	26.2
Widowed/Divorced/Separated	65	16.9
**Education**		
No education	24	6.2
Primary education	51	13.2
Secondary education	194	50.4
Post secondary education	115	29.9
**Monthly income**		
< 18 000	104	27.0
18 001–30 000	116	30.1
31 000–70 000	108	28.3
> 70 000	49	12.7

Note: For some participants, income and education level data were not provided in the questionnaire. Therefore, the total number of participants for these variables is less than the total sample size of 385.

Mean ± s.d. = 42.22 ± 10.43 (Ages: 18–30 years).

s.d., standard deviation.

### HIV profile and medical symptoms

Table 2 outlines the medical symptoms of the participants. All participants were on ART. The range of duration since diagnosis spanned from 6 months to 21 years with a median of 4, and 80% of the participants were diagnosed in the second decade of the 2000s (2011–2020). Headaches were the most commonly reported medical symptom (36.6%).

**TABLE 2:** HIV information and medical symptoms in the last 14 days (*N* = 385).

Medical symptoms	*N*	%
Abdominal pain	34	11.20
Breathlessness	17	4.40
Change in taste, sore mouth	23	6.00
Confusion	26	6.70
Diarrhoea	26	6.80
Fatigue	61	15.80
Fever	66	16.90
Headache	141	36.60
Muscular pain	65	16.90
Nausea and vomiting	16	4.20
Weight loss	107	27.80

### Health-related quality of life of participants

Table 3 shows that the mean (s.d.) for the PHS score is 54.2 ± 5.3 and MHS score is 56.3 ± 6.7. Of the 10 domains, the highest mean was found in the pain domain 57.2 ± 6.3 and the lowest mean was found in GHP domain 49.1 ± 5.1.

**TABLE 3:** HIV-related quality of life in patients with HIV – Summary scores and dimension scores.

MOS-HIV domains	Median	IQR	Mean	s.d.
**Summary scores**				
PHS	55.8	51.8–58.1	54.2	5.3
MHS	56.3	51.2–60.0	56.3	6.7
**Dimension scores**				
GHP	49.3	47.2–51.3	49.1	5.1
PF	58.1	47.2–58.1	53.5	8.4
Pain	58.3	54.5–62.2	57.2	6.3
RP	56.6	56.6–56.6	54.6	6.3
SF	57.2	57.2–57.2	53.0	10.4
MH	60.0	51.47–62.1	56.0	8.9
Energy/Fatigue	51.1	49.0–53.9	50.5	4.9
HD	70.0	53.7–70.0	56.6	8.7
CF	58.1	55.7–58.1	54.4	8.4
QoL	53.0	53.0–59.3	52.1	11.1

Note: Association between PHS and MHS showed statistical significance (Spearman’s rho = 0.243, *p* = 0.000).

IQR, interquartile range; PHS, Physical health summary score; MHS, Mental health summary score; GHP, General health perceptions; PF, Physical functioning; RP, Role-psychical; SF, Social functioning; MH, Mental health; HD, Health distress; CF, Cognitive functioning; QoL, Quality of life; MOS, Medical Outcomes Study; s.d., standard deviation.

### Factors associated with health-related quality of life

Table 4 shows that in the univariate analysis, higher education level was significantly associated with overall PHS, while being married (χ^2^ = 1.326, *p* = 0.022) and having higher income levels (χ^2^ = 14.628, *p* = 0.002) was linked to better MHS. Shorter duration of HIV was associated with better PHS (χ^2^ = 9.477, *p* = 0.009) and MHS (χ^2^ = 11.88, *p* = 0.004).

**TABLE 4:** Association between sociodemographic characteristics and physical health summary score mental health summary score.

Sociodemographic characteristics	Total (*N* = 368)	PHS				MHS			
		Bad (< 50)	Good (> 50)	χ^2^	*p*	Bad (< 50)	Good (> 50)	χ^2^	*p*
**Age (years)**	-	-	-	-	-	-	-	2.736	0.255
18–30	47	12	35	6.104	0.191	14	31	-	-
31–50	243	43	200	-	-	54	199	-	-
> 51	78	20	58	-	-	15	65	-	-
**Gender**	-	-	-	-	-	-	-	0.261	0.609
Men	101	19	82	0.211	0.646	21	83	-	-
Women	267	56	211	-	-	62	212	-	-
**Marital status**	-	-	-	-	-	-	-	1.326	0.022*
Married	209	47	162	1.326	0.515	51	163	-	-
Single	96	17	79	-	-	26	73	-	-
Widowed/Separated	63	11	52	-	-	6	59	-	-
**Education**	-	-	-	-	-	-	-	3.356	0.340
No formal	21	8	13	12.028	0.007*	6	16	-	-
Primary school	50	13	37	-	-	9	40	-	-
Secondary school	188	26	162	-	-	37	155	-	-
Post-secondary	108	28	80	-	-	31	83	-	-
**Income**	-	-	-	-	-	-	-	14.628	0.002*
₦18 000	98	20	78	0.244	0.970	33	69	-	-
₦18 001–₦30 000	113	24	89	-	-	28	85	-	-
₦30 001–₦70 000	105	21	84	-	-	14	93	-	-
> ₦70 000	45	8	37	-	-	6	43	-	-
**Duration of diagnosis (years)**	-	-	-	-	-	-	-	11.188	0.004*
1–10	294	55	239	9.477	0.009*	66	241	-	-
11–20	66	15	51	-	-	16	51	-	-
> 20	8	5	3	-	-	6	3	-	-
**PHS**	-	-	-	-	-	-	-	-	-
Bad	-	30	48	0.243	0.059	-	-	-	-
Good	-	42	241	-	-	-	-	-	-
**MHS**	-	-	-	-	-	-	-	0.243	0.059
Bad	-	-	-	-	-	30	48	-	-
Good	-	-	-	-	-	42	241	-	-

Note:

* *p* < 0.05 = statistically significant.

PHS, physical health summary score; MHS, mental health summary score; χ^2^, chi-square.

### Factors associated with health-related quality of life in the multivariable logistic regression

The results in Table 5 show that only duration of HIV diagnosis was significant in PHS and MHS in the univariate analysis and multivariate logistic regression analyses. People living with HIV who had a secondary school education showed significantly increased odds ratio (OR = 3.83; CI; 1.45–10105, *p* = 0.007) suggesting that secondary school education is associated with higher odds of better PHS HRQoL.

**TABLE 5:** Factors associated with health-related quality of life in the multivariable logistic regression (*N* = 385).

Variable	OR (95%CI)	*P*
**PHSS**		
**Education**		
No formal education (reference)	1.0	-
Primary school	1.75 (0.59, 5.18)	0.311
Secondary school	3.83 (1.45, 10.15)	0.007
Post-secondary	1.76 (0.66, 4.69)	0.259
**Duration of diagnosis (years)**		
1–10	7.22 (1.68, 31.23)	0.008
11–20	5.17 (1.21, 26.5)	0.028
> 20 (reference)	1.0	-
**MHSS**		
**Marital status**		
Married	0.32 (0.13, 0.80)	0.014
Single	0.28 (0.11, 0.74)	0.010
Widowed/Separated (reference)	1.0	-
**Income (N/month)**		
Less than ₦18 000 (reference)	1.0	-
₦18 000–₦30 000	0.29 (0.11, 0.75)	0.011
₦30 000–₦70 000	0.42 (0.16, 1.10)	0.078
> ₦70 000	0.93 (0.33, 2.58)	0.884
**Duration of diagnosis (years)**		
1–10	7.90 (1.90 – 32.5)	0.004
11–20	6.38 (1.44 – 28.4)	0.015
> 20 (reference)	1.0	-

PHSS, Physical health summary score; MHSS, Mental health summary score; OR, odds ratio; CI, confidence interval; *P, p*-value (level of significance).

Similarly, compared to the reference category of widowed or separated individuals, the results show that marital status plays a significant role, with married (95% CI: 0.13, 0.80, *p* = 0.014) and single (95% CI: 0.11, 0.74, *p* = 0.010) individuals exhibiting a substantially lower odds ratio of 0.32 and 0.28 respectively. Thus, this suggests that being married or single is associated with a reduced likelihood of experiencing poor MHS compared to being widowed or separated. Income levels also show a significant association with MHS. Individuals with an income between ₦18 000 and ₦30 000 display a notably lower odds ratio of 0.29 (95% CI: 0.11, 0.75, *p* = 0.011), indicating a decreased likelihood of poor MHS compared to those with an income less than ₦18 000.

The Hosmer–Lemeshow goodness-of-fit test yielded *p*-values of 0.989 and 0.082 for the PHS and MHS models, respectively, indicating well-fitting models. These models accounted for approximately 7% of the variability in PHS (Nagelkerke *R*^2^ = 0.077) and 10% of the variability in MHS (Nagelkerke *R*^2^ = 0.105).

## Discussion

Findings from this study revealed that the total PHS and MHS of HIV/AIDS in Lagos, Nigeria were 54.2 ± 5.3 and 56.3 ± 6.7 respectively, which were higher than those in previous studies surveyed in other sub-Saharan countries.^14,31^ Although the MOS-HIV is a widely used, reliable and validated instrument for HRQoL evaluation in PLWH,^29,32^ no parallel comparison could be drawn between our study and those conducted in other parts of Nigeria, as previous studies were conducted using other HRQoL instruments such as the World Health Organization Quality-of-Life-HIV Bref – WHOQOL-HIV BREF,^33^ Short Form-36 – SF-36,^34^ European Quality-of-Life Instrument-5 Dimension – EQ-5D.^12^ However, findings from these studies all reported good HRQoL among the participants, who were living with HIV in Nigeria. Irrespective of the measurement tool, HIV-specific HRQoL outcomes vary by country and region, with some studies showing higher overall scores than our findings.^35^ These distinctions may be ascribed to variances in healthcare infrastructure, access to treatment and cultural variables impacting perceptions of health and well-being.

Although the lowest score was reported in the health perception domain (49.3), participants in our study scored highest in MH (60.0) and HD (70.0) domains, which is similar to other studies.^36,37,38^ While several factors could be responsible for these varying results, this underscores the complex interplay between physical and MH aspects in the quality of life of individuals with HIV. One notable finding is the positive correlation (Spearman’s rho = 0.243, *p* < 0.01) between mental and PHS scores. This indicates that individuals with higher MH scores also tend to report better physical health and vice versa. This correlation emphasises the interdependence of mental and physical wellness and emphasises the significance of addressing both dimensions in interventions aimed at improving the HRQoL of PLWH.

When examining the individual dimensions of HRQoL, several trends emerge. Mental health-related dimensions, including MH, HD, and cognitive functioning (CF), show moderate to high scores, indicating relatively better mental well-being compared to physical health. Comparatively, PF and pain scores indicate moderate levels of physical well-being among participants while SF scores are notably lower, suggesting potential challenges in interpersonal relationships and social interactions. This suggests that while PLWH in Lagos may experience challenges in PF and social interactions, they exhibit resilience in coping with MH-related stressors.^39^ Several factors may contribute to health-related stress among this population.

In the present study, participants reported that they experienced headaches, weight loss and muscular pain in the previous 14 days (36.6%, 27.8%, and 16.9% respectively). These are common findings in studies conducted among the HIV population.^40^ Because of the HIV infection and adverse effects of the ART, symptoms cluster commonly reported among PLWH include depression, anxiety, insomnia, fatigue, nausea, vomiting, joint pain, and headache, all of which negatively impact the QoL among PLWH.^40^ Similarly, energy and fatigue scores (50.5 ± 4.9) indicate a substantial burden of fatigue experienced by PLWH in this population, which might impact their daily activities and overall functioning. Thus, studies that focus on fatigue management and self-perception of health promotion are needed.

The findings of this study show that education level was significantly associated with PHS. With majority (70%) of the participants educated only up to the secondary school level, the impact of education level on PLWH cannot be overemphasised. As evident from findings of previous studies,^38,41^ education level is a significant predictor of HRQoL among PLWH. Furthermore, having little formal education is a barrier to getting health treatments, leading to an escalation of unsafe sexual practices,^42^ and subsequently increasing the vulnerability of PLWH. Therefore, health interventions for PLWH should take into consideration the impact of education level and ensure that PLWH have a clear understanding of their health conditions and management strategies during hospitalisation and follow-up visits.

Duration of HIV infection was an independent risk factor that was significantly associated with the PHS and MHS of PLWH. This implies that irrespective of other confounding factors, the likelihood of experiencing physical and MH challenges or complications increases as one ages with HIV. The long-term implication of this is that policy makers and health professionals’ interventions must evolve to address both the intrinsic capacity of PLWH and the external environment.^43^

Another noteworthy sociodemographic factor in this study was that MHS was significantly associated with marital status and income level of the participants. The finding that married individuals exhibited better HRQoL compared to widowed, separated or single individuals may be attributed to several factors. Marriage often provides emotional support, companionship and a sense of belonging, which can positively impact mental well-being and overall HRQoL.^44,45^ Married individuals may also benefit from shared responsibilities, financial stability and social engagement, all of which contribute to a higher perceived quality of life. Additionally, marriage may offer a protective effect against loneliness and social isolation.^45^ Married individuals have access to greater social support networks and resources, which can buffer against the negative effects of living with HIV and associated challenges.

Furthermore, our study findings suggest that individuals with an income between ₦18 000 and ₦30 000 have a significantly lower likelihood of experiencing poor MHS compared to those with an income less than ₦18 000. The odds ratio of 0.29 indicates that individuals in the higher income bracket have a substantially reduced risk of poor MHS. With over half (59.3%) of the participants in our study earning less than ₦30 000 ($65.07) monthly, which is the minimum wage in Nigeria,^44^ the socioeconomic impact of HIV cannot be overlooked.

Understanding the socioeconomic factors associated with HIV allows policymakers to develop more effective and inclusive policies aimed at mitigating disparities and improving outcomes for affected populations. Moreover, by acknowledging the socioeconomic determinants of health, healthcare providers can offer holistic care that addresses not only the medical aspects of HIV but also the social and economic factors that influence health outcomes.^45^ This may involve connecting patients with social support services, financial assistance programmes, and vocational training opportunities to enhance their overall well-being and quality of life. By addressing the underlying social and economic factors that contribute to HIV-related disparities, healthcare professionals and policymakers can work towards achieving better health outcomes and promoting equity in healthcare access and delivery. Thus, further research is needed to understand potential interventions to improve quality of life and address confounding socioeconomic disparities among affected populations.

### Study limitations

Nearly one-third of the participants reported experiencing weight loss during the 14-day period. However, as the study did not include objective measurements of weight before and after this timeframe, it is challenging to accurately quantify the extent of weight loss. This lack of objective data underscores a broader limitation inherent in self-report cross-sectional studies, where reliance on participants’ subjective accounts may introduce inaccuracies or biases. Another limitation was that the study was conducted only in Lagos state. Future studies need to be conducted in other HIV/AIDS ‘key population hotspots’ in Nigeria to determine the variation in quality-of-life scores across geographic areas. Also, the cross-sectional design of our study prevented us from establishing the precise causality between HRQoL and its associated factors. Consequently, further research aimed at exploring causal relationships between these variables is vital.

## Conclusion

Our study revealed that the HRQoL of PLWH in Nigeria is relatively low. The findings highlight the importance of addressing the multifaceted determinants of HRQoL among PLWH in Nigeria. By understanding these determinants, healthcare professionals, policymakers and stakeholders can work together to improve the quality of life for PLWH, thereby enhancing their health outcomes and promoting a more inclusive and supportive environment for individuals living with HIV.
